# Wound Infection After Cleft Lift Operation for Pilonidal Disease

**DOI:** 10.7759/cureus.77140

**Published:** 2025-01-08

**Authors:** Steven C Immerman

**Affiliations:** 1 Surgery, Evergreen Surgical, Eau Claire, USA

**Keywords:** bascom cleft lift, cleft lift procedure, cleft lift surgery, pilonidal, pilonidal surgery, postoperative wound infection

## Abstract

Objective

This is an analysis of postoperative wound infections that developed in patients who had the cleft lift procedure for both primary pilonidal disease and for salvage after previous other failed operations. Infection after the cleft lift procedure can complicate or prolong healing and recovery by several weeks. The aim of this report is to help clinicians recognize and effectively manage these patients.

Patients and methods

All patients who had the cleft lift procedure by the author and developed postoperative infection during the period between January 2014 and May 2024 were included. This study evaluates the intraoperative measures to prevent infection, describes clinical presentations, and outlines specific treatment strategies for postoperative infections.

Results

During the approximate 10-year time period of this study, there were 1,200 patients who had the Bascom cleft lift procedure, and within that group, there were 39 patients who developed wound infections, for an overall infection rate of 3.3%. All patients with diagnosed postoperative infection were prescribed additional antibiotics, and if drains were present, they were left in place until the infection had clinically subsided, as was the case for 19 patients (49%). Two patients (5%) required return to the operating room (OR) for irrigation of the wound and replacement of drains, and three patients (8%) required placing additional supporting sutures in the outpatient clinic. 15 patients (38%) had their drain already removed when the infection became apparent, and they were successfully treated with antibiotics. Ultimately, 37 of the 39 patients went on to complete healing without the need for cleft lift revision, demonstrating the effectiveness of timely intervention. Two patients were lost to follow up.

Conclusions

Infections should be recognized and treated quickly with adjustment of the antibiotic regimen and assurance of adequate drainage. Maintaining the position of the lower incision is of paramount importance, and if these infections are properly treated, they do not jeopardize the ultimate success of the operation.

## Introduction

The cleft lift operation for pilonidal disease is an operation that was first described by Dr. John Bascom in 1987, and at this time, it is considered by many surgeons to be the optimal surgical procedure for the treatment of pilonidal disease [1-3]. Although various surgeons have described modifications of the technique, the basic concepts of flattening the gluteal crease, removing active disease, and positioning the incision off-midline are commonalities [4-6]. As with any operation, wound infections do occur and can cause serious problems including wound separation and predisposition to recurrent pilonidal disease. The published incidence of wound infections after a cleft lift procedure varies from 1% to 13.2%, but there has not previously been a report discussing the presentations and treatments of surgical site wound infections as they specifically pertain to the cleft lift operation [3,7]. This article discusses an approximate 10-year experience with infections after the cleft lift procedure in a busy pilonidal practice. Patterns of presentation of these infections and treatment strategies are discussed, along with their effect on ultimate healing and recurrence rate.

## Materials and methods

This is an analysis of all patients who had wound infections after a Bascom cleft lift by the author during the time period from January 2014 to May 2024. Clinical information was contemporaneously entered into a database at the time of surgery, and follow-up was gleaned from clinic visits, the electronic medical record, patient emails, and patient surveys. The minimum follow-up time was six months for the most recent patients and as long as six years for the early patients. Two patients were lost to long-term follow-up, but were each followed for at least one year. The details of the cleft lift operation, as performed by the author, are provided in a previous publication, but the specific interventions that pertain to preventing and treating infection are described further, which include the use of skin preparation, intraoperative wound lavage, antibiotics, dressings, and drain placement [8].

Intraoperative routine

After positioning the patient prone on the operating table, the buttocks were taped apart, clipped of hair, scrubbed with providone iodine soap, and then painted with providone iodine antiseptic. Intravenous antibiotics were administered in the operating room (OR) immediately before the incision. In 35 patients, the antibiotics consisted of a fluoroquinolone plus metronidazole. The remaining patients received ampicillin/sulbactam (three patients) or clindamycin (one patient) because of allergy or patient preference due to previous side effects. Once the contaminated specimen was removed, and prior to closure of the deep layer, all wounds and surrounding skin were irrigated with multiple manually-pulsed, irrigating bulb syringes filled with Clorpactin™ antiseptic solution, using approximately 750 cc of lavage solution in an attempt to clean the operative site from any contamination that may have occurred during manipulation of the cyst or sinus. Intraoperative cultures were only taken if actual purulence was encountered during the procedure. Surgical gloves were changed at this point in the procedure if open wounds or draining sinuses had been encountered.

All patients had a 15 French closed suction drain placed directly under the rotation flap and the plan was to leave this in place until postoperative day 5-7, and remove it if the drain output was not purulent or cloudy and less than 20cc/24-hour period. The deep layers and skin were infiltrated with liposomal marcaine to provide postoperative analgesia for 72 hours.

**Figure 1:**
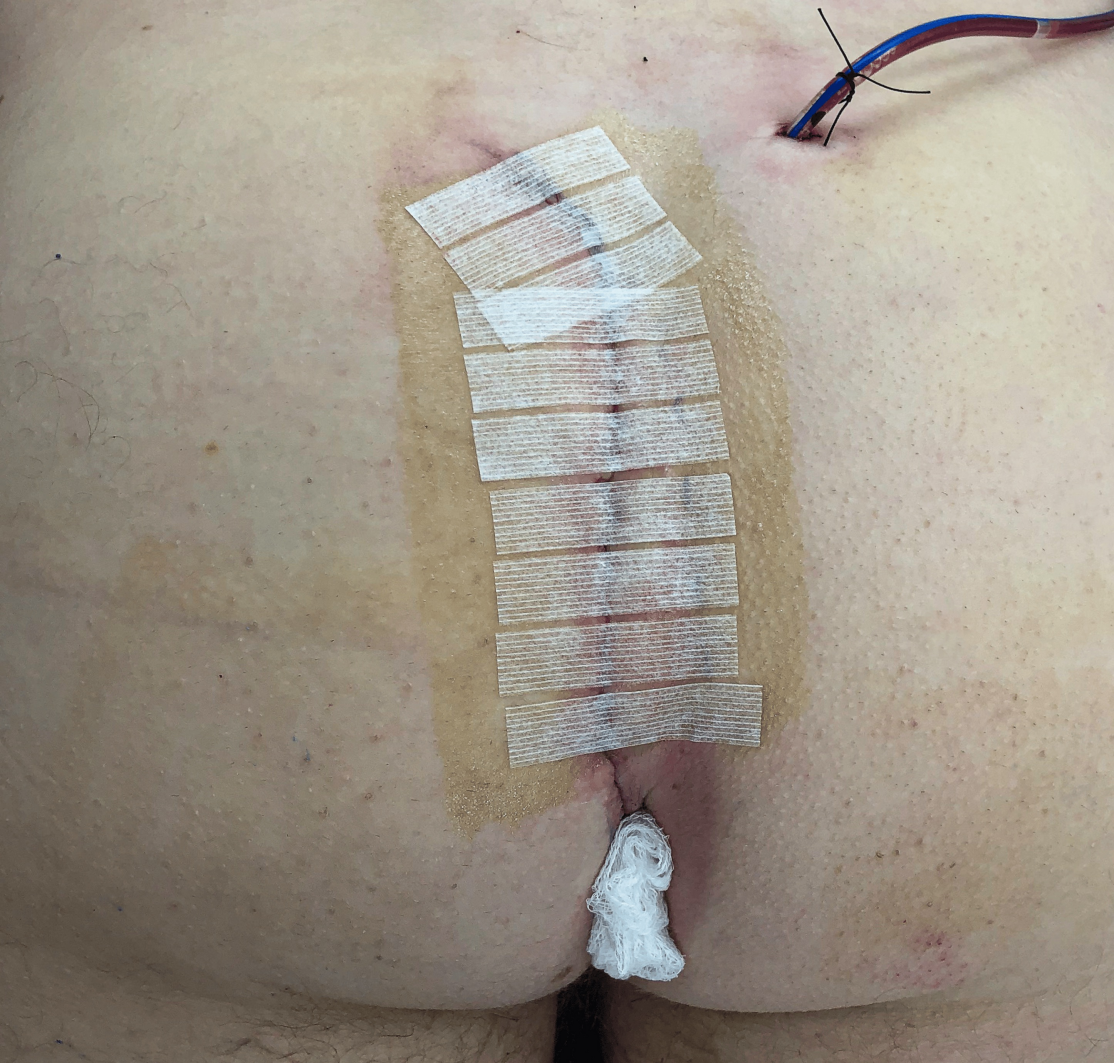
Appearance of drain and perianal gauze placement after a completed Bascom cleft lift procedure

All wounds were closed with absorbable subcuticular sutures. Steri-Strips™ were placed perpendicularly across the upper three-quarters of the incision; no Steri-Strips™ were placed over the perianal portion of the incision. Postoperative dressings consisted of gauze dressings over the incision and drain insertion site, with a folded piece of woven gauze placed next to the perianal portion of the incision to improve aeration (Figure 1). Surgical glue was not utilized.

Postoperative care

All patients were prescribed oral antibiotics for one week, to be started the evening of surgery. These consisted of either a fluoroquinolone+metronidazole, ampicillin/clavulanate, or clindamycin.

After discharge, the upper dressings were changed daily until dry, and then discontinued; the perianal gauze was changed twice daily for one month. No routine topical medications were applied. Patients were encouraged to shower daily starting with the first postoperative day, but asked to avoid prolonged soaking of the incision for one month.

Dealing with infection

The determination that a wound infection was present was always made by the author either by direct physical examination or examination of photos of the incision and drain collection reservoir. Any increase in pain made early infection suspect, and erythema, drainage through the wound, or purulent drainage in the closed suction drain bulb would confirm the diagnosis. If infection was suspected, the drain was left in place, antibiotics were changed, and the length of antibiotic therapy extended until clinical indications demonstrated that the infection had resolved, as evidenced by the resolution of the pain, erythema, and purulent wound or suction drain output.

## Results

During the study period the Bascom cleft lift was performed on 1,200 patients and of these, 39 patients were determined to have a wound infection. Of this cohort, 74.4% were male, and 25.6% were female, with an average age of 25 years. Two-thirds of the patients (67%) had been through previous excisional pilonidal surgery that failed (Table 1).

**Table 1:** Cohort characteristics. Age 25 ± 8.8 years.

	Number	Percentage
Male	29	74.4%
Female	10	25.6%
Previous Surgery	26	67%

The preoperative presenting findings were sinus tracts in 16 patients (41%), open wounds in 12 patients (31%), recurrent abscesses in 9 patients (23%), an inflamed sacral dimple in 1 patient (2.5%), and a painful scar with retained foreign material in 1 patient (2.5%) (Table 2).

**Table 2:** Presention of patients who developed infection

	Number	Percentage
Sinus	16	41%
Wound	12	31%
Recurrent Abscesses	9	23%
Sacral Dimple	1	2.5%
Painful Scar	1	2.5%

44% of patients had wounds or sinus tract openings in the perianal region, and of these, two patients had wounds directly at the edge of the anoderm. Six patients had diagnosed or suspected hidradenitis suppuritiva. A comparison of the patients with wound infections to the entire 1,200-patient cohort is shown in Table 3.

**Table 3:** Comparison of the 1,200-patient cohort to patients who developed wound infection

	Patients with infections (N=39)	Total cohort (N=1200)
Male	29 (74%)	817 (68%)
Female	10 (26%)	383 (32%)
Previous Surgery	26 (67%)	580 (48%)
Perianal Wounds	17 (44%)	491 (41%)
Wounds at the Edge of Anoderm	3 (5%)	114 (12%)
Hidradenitis Suppuritiva	6 (15%)	35 (3%)

There were no statistically significant differences between the patients with infections as compared to the total cohort *(P*>0.05). If the original presenting symptoms were analyzed in all 1,200 patients, we found that the infection rate in patients who presented with open wounds was 3.8%, with sinus tracts 2.7%, and with repeated abscesses 3.1%, suggesting that the presentation was not a contributing factor to the development of infection. There was a higher proportion of patients in the infection group who had previous excisional surgery or had hidradenitis suppuritiva, suggesting that these may be risk factors, though this does not reach statistical significance in this cohort of patients.

Only one patient in the group with infection had an intraoperative culture sent. It grew actinomyces and the patient was treated with three months of ampicillin/sulbactam.

**Figure 2:**
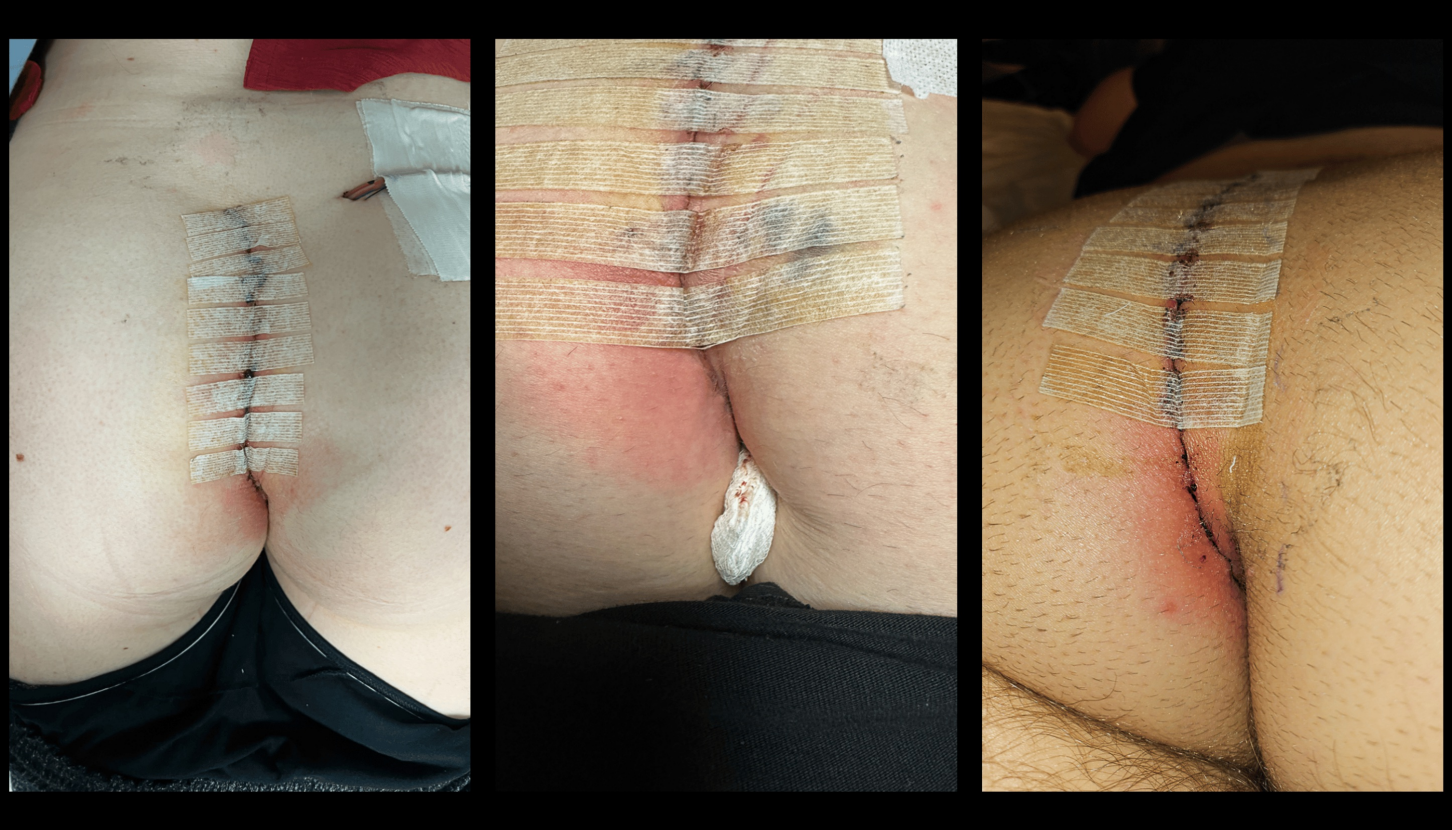
Examples of cellulitis developing toward the inferior aspect of the incision in the first postoperative week

**Figure 3:**
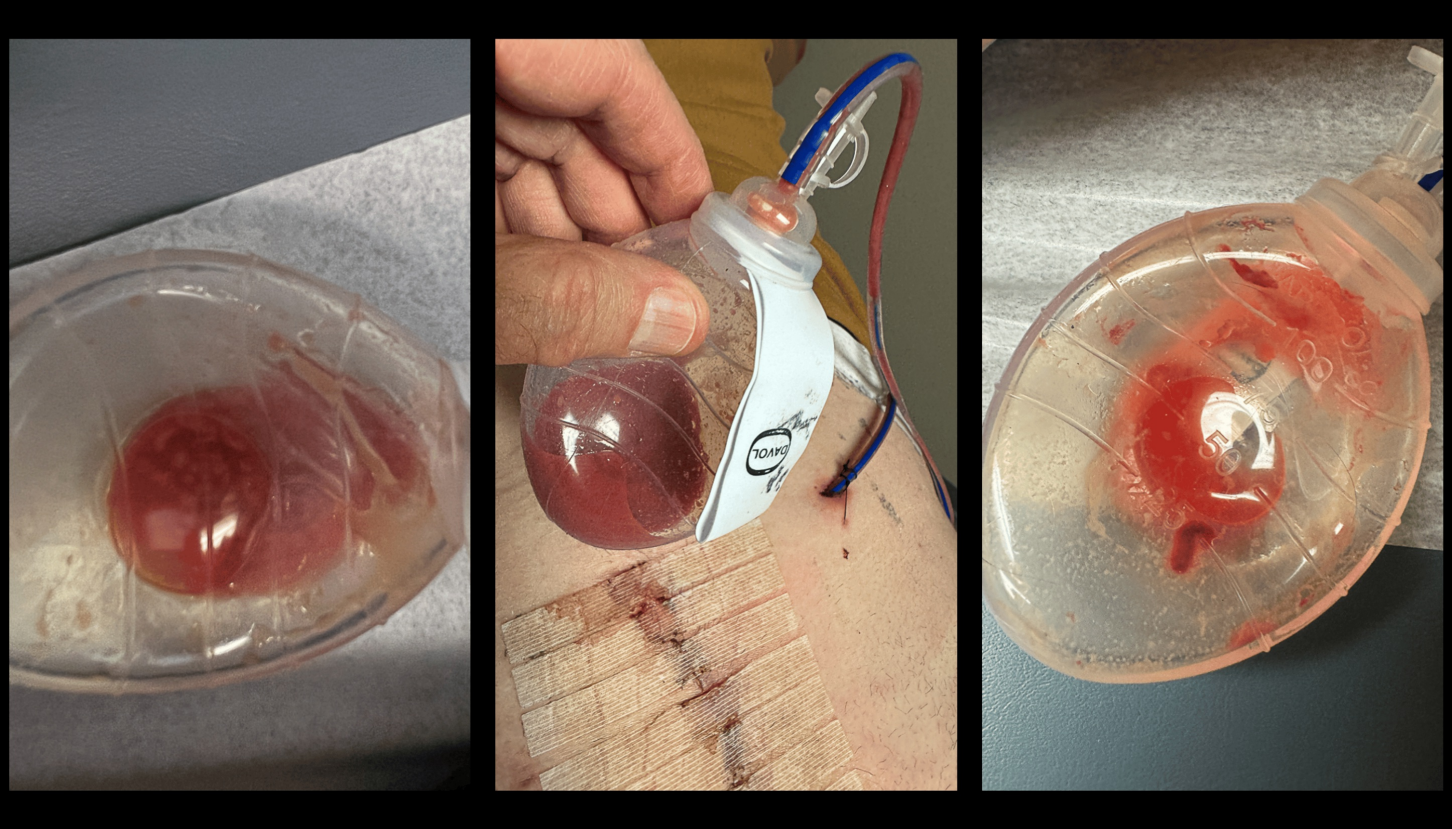
Purulent suction drain output

The wound infections became clinically evident in several fashions, the most common of which was cellulitis in the inferior portion of the incision, often on the non-flap side (Figure 2). Other presentations were purulent drainage from the closed suction drain (Figure 3), drainage through the incision, or infected hematoma (Table 4). Often, the first sign of infection was increasing pain in the lower incision around postoperative days 3-5.

**Table 4:** Clinical presentation of postoperative wound infection

Presentation of Infection	Number of Patients
Cellullitis	20 (51%)
Drainage through Incision	11 (28%)
Purulent Suction Drain Output	7 (18%)
Infected Hematoma	1 (3%)

**Figure 4:**
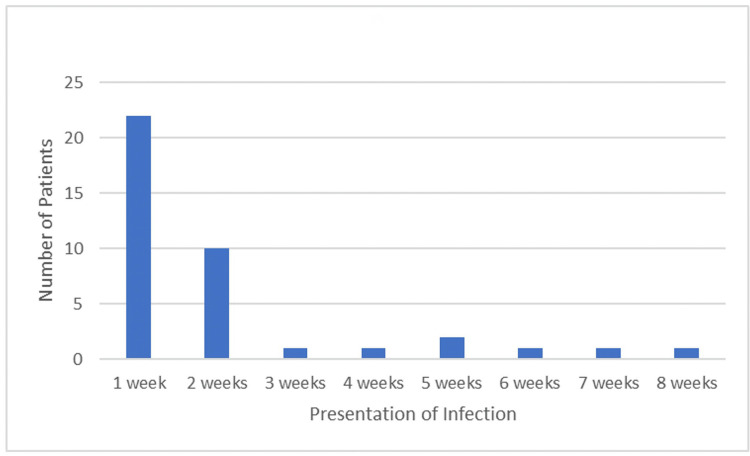
Timing of presenting symptoms of infection

Most infections became evident within the first seven days from surgery in spite of all patients being on oral antibiotics at that time (Figure 4).

**Figure 5:**
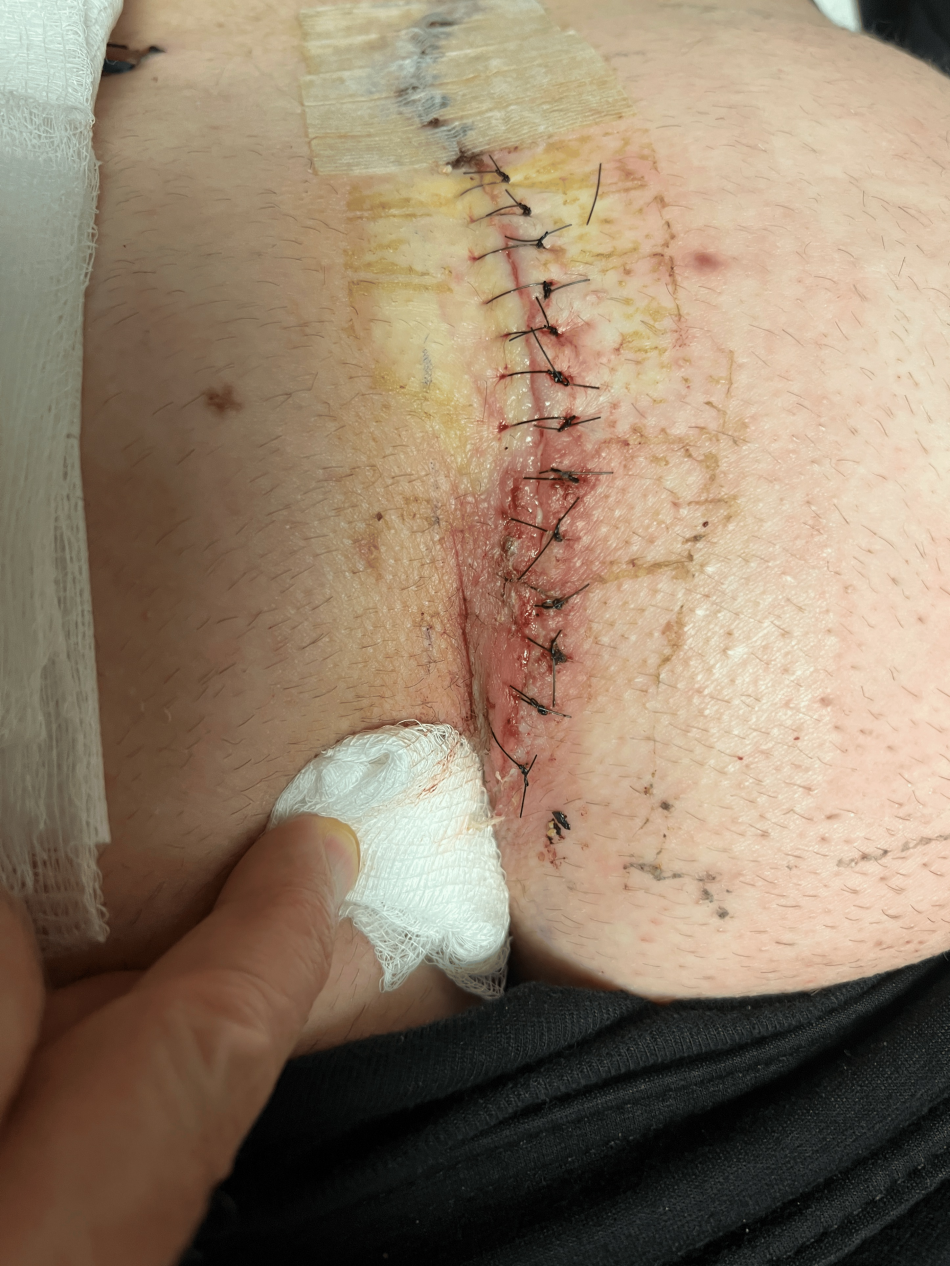
Sutures placed postoperatively to prevent separation of the lower incision

In 19 patients the infection was treated by leaving the drain in place until the infection had clinically resolved and adjusting and prolonging antibiotic therapy. In two patients, the drain did not seem to be adequate, and the patients were returned to the OR to open the upper portion of the incision, lavage the wound with Clorpactin™, replace the 15F drain with a 19F drain, and re-close the incision with external nylon sutures. In three patients, an incipient wound separation at the lower third of the incision prompted placement of external nylon sutures in the outpatient clinic to prevent a significant dehiscence of this portion of the wound while the drain was still in place. In 15 patients, the drain had been removed by the time the infection was diagnosed, presenting with either cellulitis or drainage through the incision after the first postoperative week. These patients were successfully treated with antibiotics alone. Because they did not have wound separation to a degree that would jeopardize the success of the cleft lift, drain re-insertion or adding additional sutures was not felt to be necessary, and the successful clearance of the infection substantiated this decision (Table 5). An example of sutures placed to prevent wound separation is shown in Figure 5.

**Table 5:** Treatment of infections OR: Operating room

Treatment of Infection	Number of patients
Leave Drain + Antibiotic Change	19 (49%)
Drain Already Out, Additional Antibiotics	15 (38%)
Leave Drain + Antibiotic Change + Add Sutures to Lower Incision	3 (8%)
Antibiotic Change + Return to OR for Drain Replacement	2 (5%)

## Discussion

Differentiating infection from dehiscense

It is important to differentiate an actual wound infection in postoperative cleft lift patients from a dehisced and subsequently contaminated wound. If the flap is improperly constructed such that the incision is in the midline, and when the buttocks are compressed it folds directly on the incision, it is not unusual for this wound to separate, sometimes very quickly and dramatically after surgery. This is often attributed to a wound infection when that is not actually the case; it is rather a failure of the operation to obtain the proper contour and a subsequent dehiscense. In the patients presented in this series, the cleft lift flap was positioned off-midline, and these patients did not primarily have wound dehiscence but actual postoperative wound infections in closed wounds.

**Figure 6:**
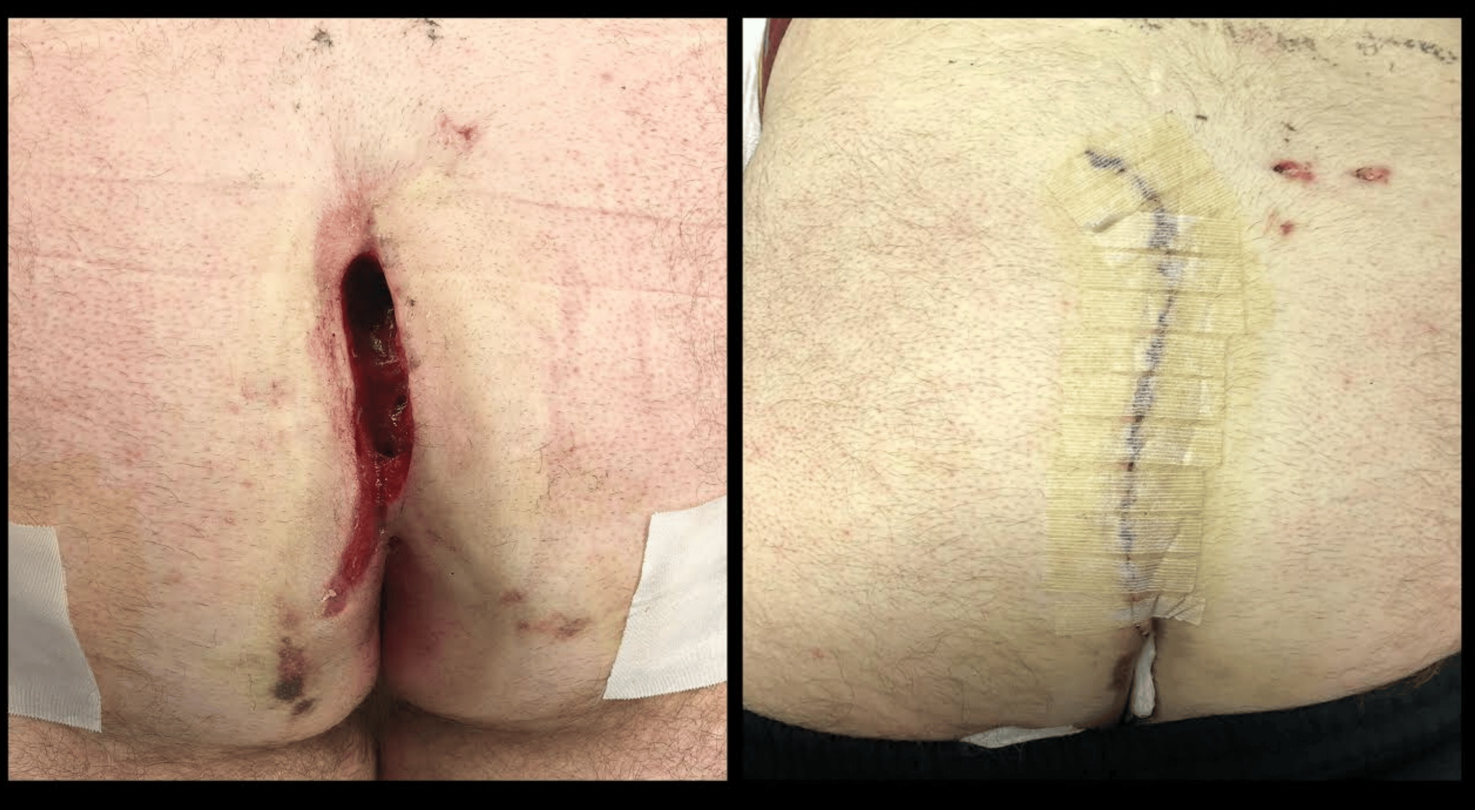
Failed cleft lift attributed to infection on left; successful revision on right

Figure 6 demonstrates an example of this situation. This is a 22-year-old male who had a cleft lift procedure at another clinic, which left him with a midline incision. By one week post operation, this began separating, and this was attributed to infection. The entire wound was then opened to drain the perceived infection. The patient was seen at three weeks postoperatively and the cleft lift was revised such that the incision was distinctly off-midline. He went on to heal primarily and did not develop recurrent disease during the following five years.

Antibiotics

We select specific prophylactic antibiotics in an attempt to cover the broad spectrum of bacteria with which pilonidal cysts and sinuses are commonly colonized. An analysis of the bacteria found in these lesions was done by Brook and published in a 1989 article that demonstrated only anaerobic bacteria in 77% of patients, only aerobic bacteria in 4%, and mixed aerobic/anaerobic flora in 19% [9]. This would suggest that coverage of anaerobic bacteria is of importance when using prophylactic antibiotics, and common choices would be metronidazole, ampicillin/sulbactam, or clindamycin. In order to cover the full spectrum of organisms that commonly inhabit these lesions, coverage of gram positive and gram negative aerobes is also reasonable, and a flouroquinolone, cephalosporin, or amoxicillin/clavulinate are appropriate choices. However, whether postoperative prophylactic antibiotics are necessary at all is somewhat controversial. In Dr Brian Shrager’s 2023 article, he describes a 4.7% infection rate without using routine prophylactic antibiotics postoperatively except in acutely infected cases [10]. We continue to use them because of our low infection rate, and high overall success rate, but whether their use in all patients is necessary is a topic for further research.

Because intraoperative cultures were rarely available, antibiotic strategies were usually empiric once an infection became evident. Postoperative cultures in patients demonstrating infection were only obtained in situations where the infection seemed particularly aggressive or unusual in some way. The reason for this was that changing antibiotics was felt to be urgent, and had to be performed well before culture and sensitivity results could be obtained. Dramatic findings in three patients did prompt obtaining cultures that grew methicillin-resistant *Staphylococcus aureus* (MRSA). These were all sensitive to sulfamethoxazole and trimethoprim.

During this 10-year period, differing strategies were used to guide the empiric change in antibiotic selection in patients with infection. Initially, the strategy was to extend the same antibiotic for another week. As experience was gained with treating these postoperative infections, we found that the best strategy was to change to antibiotics that might have a slightly different spectrum of coverage. Our experience with several patients’ cultures that unexpectedly grew MRSA prompted the use of sulfamethoxazole and trimethoprim as a second-line antibiotic along with ampicillin/sulbactam, and this combination has proven quite effective in quickly improving the situation; so effective that we rarely do cultures even with clinical signs of infection. We consider this change in antibiotic coverage to be emergent, and if patients contact us with increasing pain or erythema, we ask them to email us photos of the incision and the drainage fluid, and if infection is suspected, a new antibiotic prescription is electronically sent to the pharmacy immediately; we do not feel that this can wait until a clinic visit the next day. The patients all have the author's email address, so these concerns can be addressed after hours or on weekends. This prompt recognition and treatment of early infection will often prevent further problems.

Drain placement

We routinely place a 15F closed suction channel drain in all patients. This drain is placed after closure of the deep layer of subcutaneous tissue over the sacrum and coccyx, and inserted under the rotation flap. Another option used by many surgeons is to place a passive drain, but we have not utilized that modality. Our logic is that in cases of infection, significant seroma, or hematoma the small, passive drain may be inadequate. We have found that the postoperative care of a closed suction drain has been quite manageable for our patients. However, the necessity of a drain in all patients is something that has been questioned. An article by Gurer et al. from 2005 studied this as it applies to patients having the Karydakis flap procedure which is similar to the cleft lift, and they found a 20% complication rate in patients without a drain, and that "the complications were caused exclusively by fluid collection" [11]. Faruschou et al. compared the utilization of closed suction drains for seven days with passive drains for one day, and did not find any difference in wound healing, but they do not specifically comment on the incidence of post op seroma, hematoma, or wound infection [12]. The value of a drain may also vary depending on the surgeon's technique, particularly as it pertains to the width of the rotation flap. This is also a topic for further research.

Once a drain is in place, it is important that the surgeon be suspicious in looking for subtle signs of early infection such as increasing pain, cloudy drain output, and mild erythema. It would be easy to attribute any increase in pain around postoperative day three to the loss of analgesic effect from the liposomal marcaine, but we have not found that patients complain of increasing pain as the local anesthetic wears off, except in cases of infection. Any complaints of increasing pain should be considered a sign of early infection until proven otherwise, and any subtle signs of infection should prompt the consideration of leaving the drain in longer and changing antibiotics. As this series of patients progressed, this was a lesson we learned, and our threshold for taking these steps decreased.

Intraoperative wound lavage

For this series of patients, the wound was irrigated intraoperatively after all contaminated tissue was removed from the operative field. The irrigation included all areas of exposed skin, the anus, and the wound itself in an attempt to attain sterility prior to closure. Surgeons have several options for the lavage solution including saline, antibiotics in saline solution, or an antiseptic. Our chosen lavage solution is sodium oxychlorosine (Clorpactin™ WCS-90, United Guardian Inc, Hauppage, NY, USA). This is a hypochlorous acid derivative that is extremely bacteriocidal to a broad spectrum of organisms and has a pH of 6.8-6.9 [13]. It is immediately ready for use after mixing 2 grams of powder in 1 liter of warm saline in a container, and stirring for a few moments. At our facility the cost to the patient for this antiseptic is $37. In 2021 Alentado et al. published a study using Clorpactin™ as an intraoperative irrigant in 1,043 patients undergoing spine surgery. They found a decrease in infection rate from 3.5% to 1.2% with no adverse effects, suggesting that it is a safe and effective intraoperative wound lavage solution [14]. Our empirical selection of a lavage solution that was highly bacteriocidal, easy to prepare, gentle on tissues, and economical, has driven our choice of this product. There are no published studies comparing the use of Clorpactin™ to other irrigants during the cleft lift procedure. This is an empirical choice, and further research into the optimal lavage solution would be helpful to surgeons.

Wound management

The ultimate goal in these patients is to have the cleft lift heal completely while maintaining the position of the incision and shape of the re-contoured cleft. If significant wound dehiscence of the lower portion of the incision occurs because of an infection, it is possible that the wound will migrate to the midline and become a chronic-non-healing wound or a nidus for future sinus tract development. For this reason, opening these wounds and allowing them to heal secondarily is contraindicated in these patients, which is in opposition to the standard treatment for wound infections in other situations. For cleft lift patients the drain is either left in place longer; replaced if removed; replaced if not functioning adequately; or the closed wound managed with antibiotic therapy. In cases of significant wound separation, placing external, interrupted, nylon sutures has proven effective in supporting the wound and preserving its position. These can be removed once the infection has resolved.

Incidence of Infection

Not all series of cleft lift patients report an infection rate, and often wound infection and dehiscense are combined, or there is confusion regarding these two problems. However, several studies do report a specific rate of infection: Agcaoglu et al. describe a 10% infection rate in 60 patients, Hatch et al. describe a total wound infection rate of 10.6% in a series of patients with an overall complication rate of 50%, Tezel et al. describe a 13.2% infection rate in 76 patients and specifically differentiate wound dehiscense from infection, and Svarre et al. report 200 patients from Copenhagen which reveal an 8.1% infection rate [7,15-17]. As previously mentioned, Shrager's 4.7% infection rate without the use of postoperative antibiotics is interesting and requires further study [10]. Our 3.3% rate of infection compares favorably and the reason for this is unclear, but may represent differences in surgical technique as well as our infection prevention regime. This infection rate of 3.3% is also demonstrated in a series of 30 Bascom cleft lift patients reported by Karim et al. in a 2020 paper from Great Britain [18]. A similar 3.4% infection rate is reported in a series of 33 patients from Norway in the paper by Rushfeldt et al. [19].

Limitations of this study

This study is limited by the fact that all procedures were done by a single surgeon. The variations in exactly how this procedure is performed by different surgeons may effect the incidence of infection, how it presents, and the ultimate consequence of the infection on the success of the procedure. Further study is needed to determine the necessity of prophylactic antibiotics in all patients, the optimal wound lavage solution, and the necessity of suction drain placement in all patients.

## Conclusions

As more surgeons become aware of the cleft lift technique, it is inevitable that infections will be increasingly encountered in clinical practice. Because these infections are uncommon, it takes experience with many patients to begin to see patterns and learn treatment strategies. There are lessons to be learned from this cohort of patients with postoperative infection that can be useful to others, and the patients towards the end of the series benefited from the lessons we learned earlier in the study period. If these infections are recognized quickly and treated appropriately, these patients go on to heal completely, and the presence of the postoperative infection does not ultimately predispose to recurrent pilonidal disease or failure of the cleft lift incision to heal.

Several of the hallmarks of effective recognition and treatment of these infections are non-intuitive. These include understanding that: (1) These infections often occur during the first postoperative week, even though patients are still on antibiotics; (2) Pain is often the first sign of infection and should prompt immediate evaluation; (3) These infections usually occur in the perianal end of the incision; (4) Cultures, while occasionally helpful, are rarely needed to manage these patients; (5) Rapid adjustment of the antibiotic regimen, including adding sulfamethoxazole and trimethoprim along with ampicillin/sulbactam, will often turn these infections around quickly; (6) It is contraindicated to open the perianal portion of an infected wound to allow it heal secondarily, but rather it is best to add supporting sutures to keep this portion of the incision closed while keeping the closed suction drain in place; (7) The initial presentation of pilonidal disease does not have an effect on the incidence of infection; and (8) Patients with previously failed surgery or hidradenitis suppuritiva may have a higher incidence of wound infections and should be observed closely.
